# Supplementary material for: Structural basis of hydroxycarboxylic acid receptor signaling mechanisms through ligand binding
Source: Nat Commun. 2023 Sep 22;14:5899. doi: 10.1038/s41467-023-41650-7 (PMC10516952; doi:10.1038/s41467-023-41650-7)
Supplement: Supplementary file 1 — Supplementary Information [file 41467_2023_41650_MOESM1_ESM.pdf]

Supplementary Information
for
Structural basis of hydroxycarboxylic acid receptor signaling mechanisms through ligand binding

Suzuki S et al.

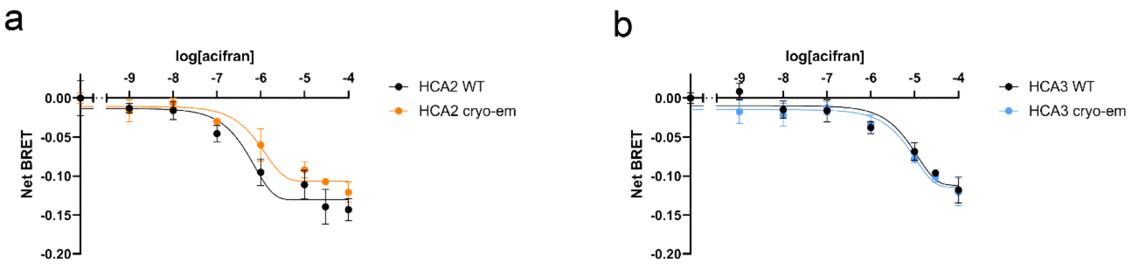

Supplementary Fig. 1 | Functional validation of the constructs for structural studies

a,b Dose-response curves of Gαi–Gγ dissociation induced by acifran via HCA2 (a) and HCA3 (b) in HEK293 cells overexpressing wild-type (WT) or thermostabilized cytochrome b562RIL (BRIL)-fusion constructs (cryo-EM). Data from 3 independent experiments are presented as the mean ± SEM (n=3).

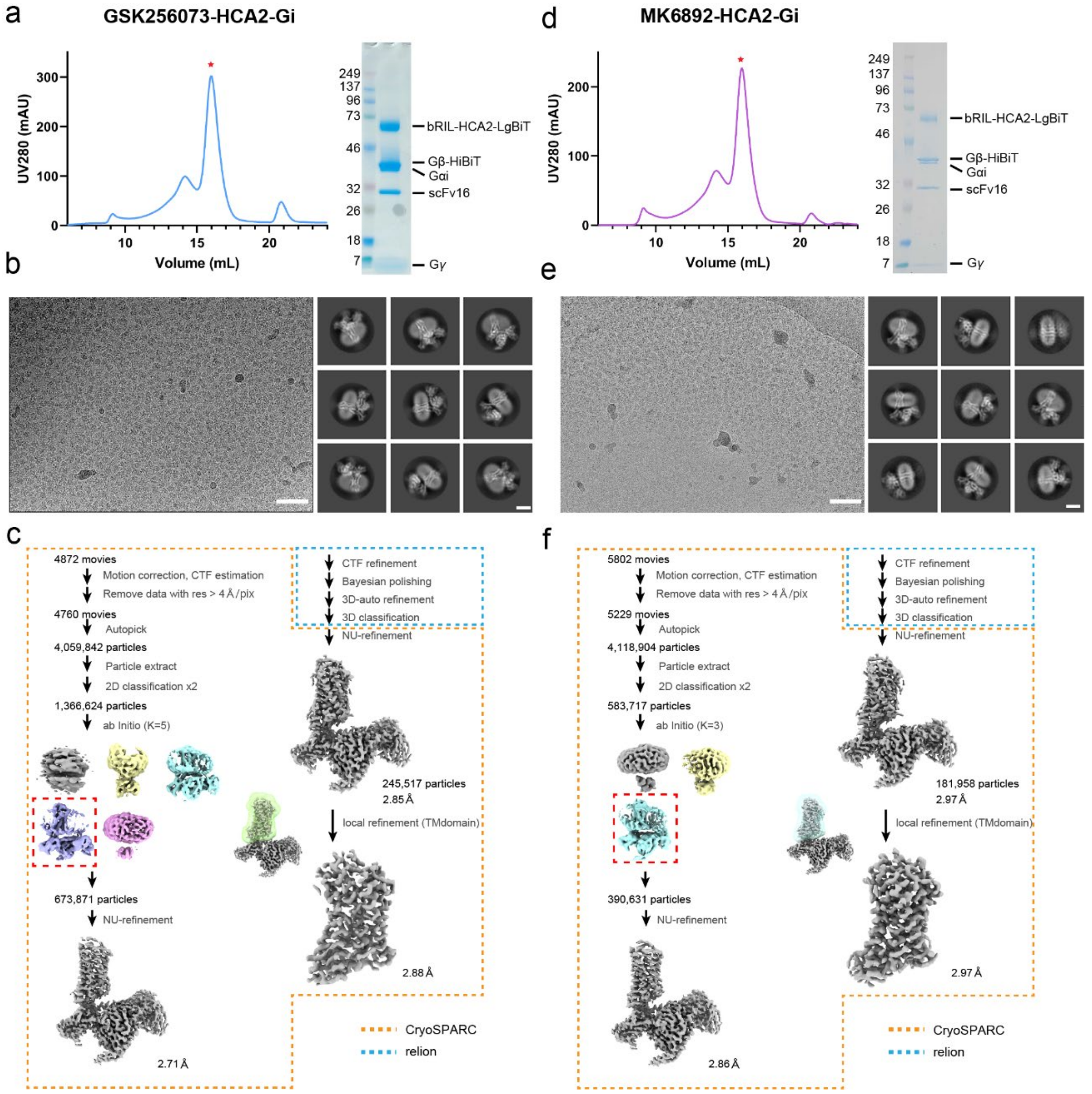

Supplementary Fig. 2 | Cryo-EM sample preparation and data processing for the GSK256073 and MK6892-bound HCA2 complexes

a,d Representative elution profile of *in vitro* reconstituted HCA2–Gi complex on a Superose 6 Increase 10/300 column and SDS-PAGE of the peak fraction (red asterisk). **b,e** Representative micrograph and 2D class averages (scale bars indicate 20 nm and 5 nm, respectively) **c,f** Flow chart of cryo-EM data processing of the HCA2–Gi complex.

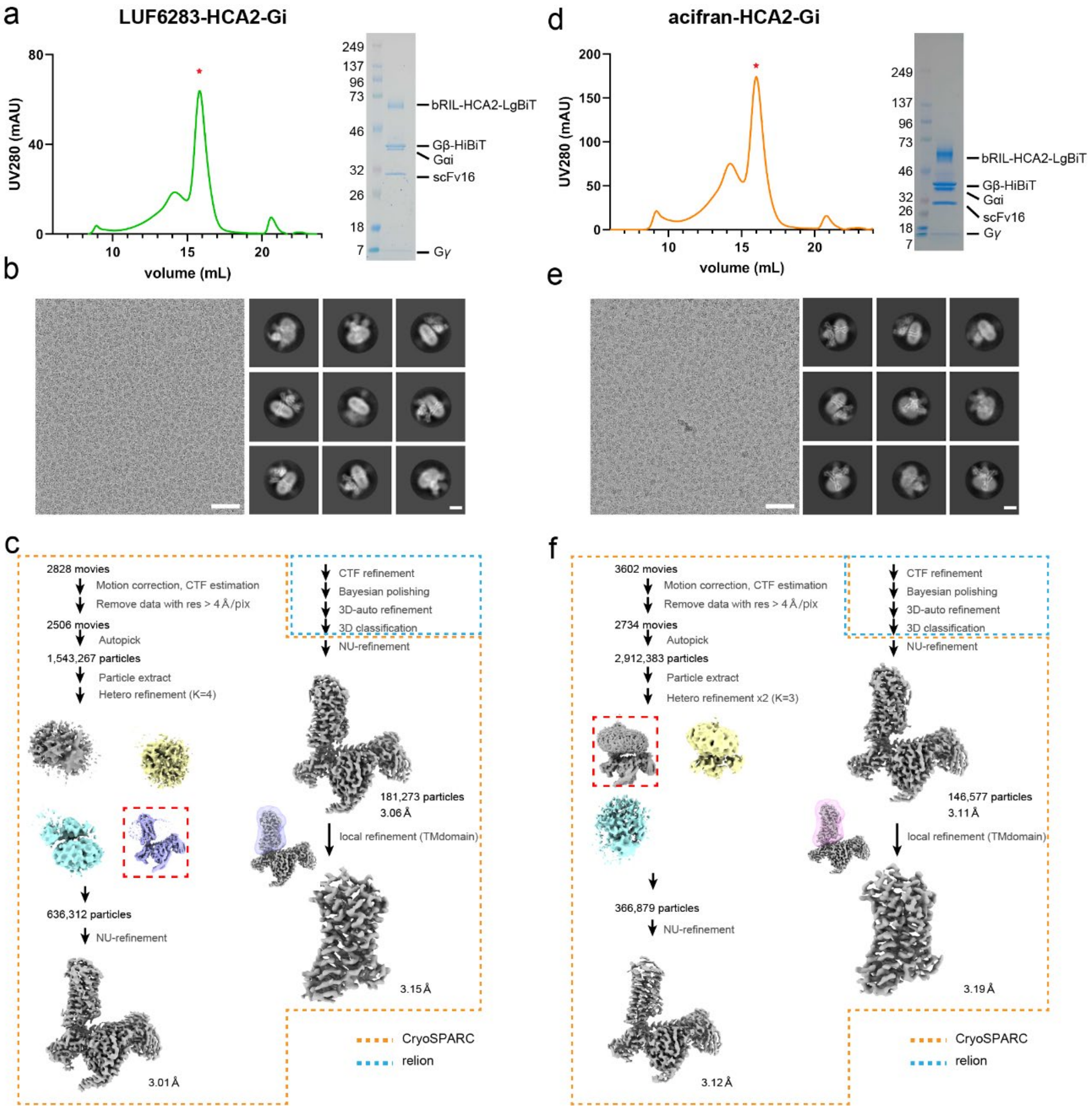

Supplementary Fig. 3 | Cryo-EM sample preparation and data processing for the LUF6283 and acifran-bound HCA2 complexes

a,d Representative elution profile of *in vitro* reconstituted HCA2–Gi complex on a Superose 6 Increase 10/300 column and SDS-PAGE of the peak fraction (red asterisk). **b,e** Representative micrograph and 2D class averages (scale bars indicate 20nm and 5 nm, respectively). **c,f** Flow chart of cryo-EM data processing of the HCA2–Gi complex.

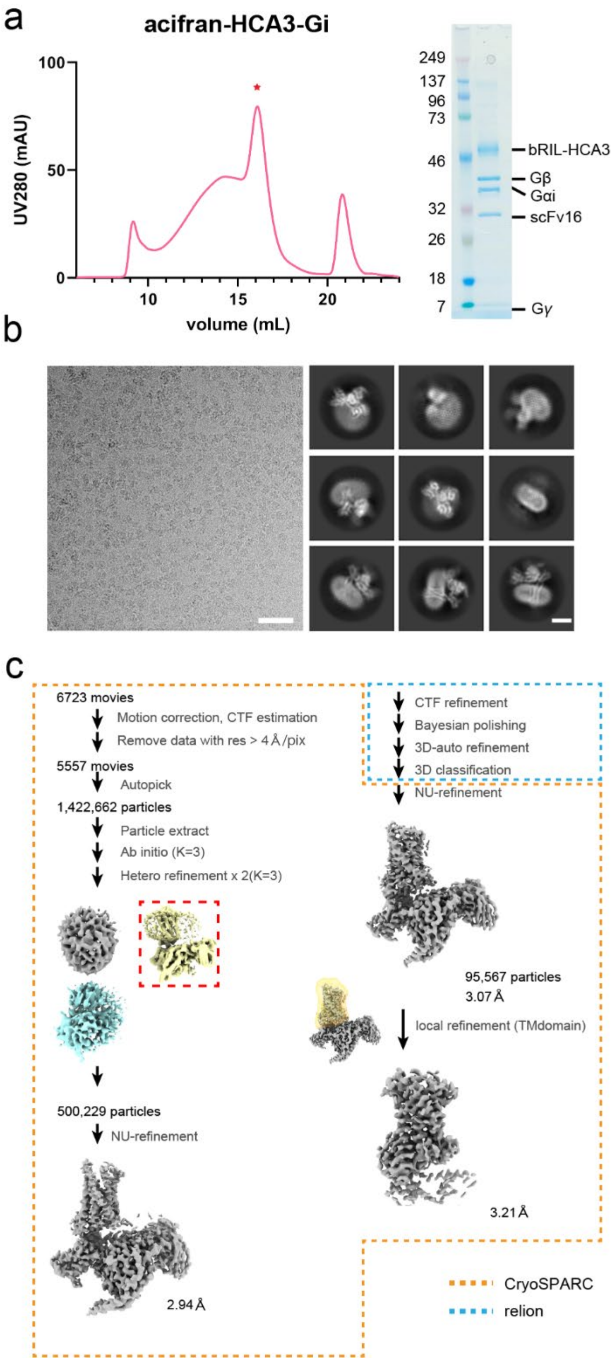

Supplementary Fig. 4 | Cryo-EM sample preparation and data processing for the acifran- bound HCA3 complex

a Representative elution profile of *in vitro* reconstituted HCA3–Gi complex on a Superose 6 Increase 10/300 column and SDS-PAGE of the peak fraction (red asterisk). **b** Representative micrograph and 2D class averages (scale bars indicate 20 nm and 5nm, respectively). **c** Flow chart of cryo-EM data processing of the HCA3–Gi complex.

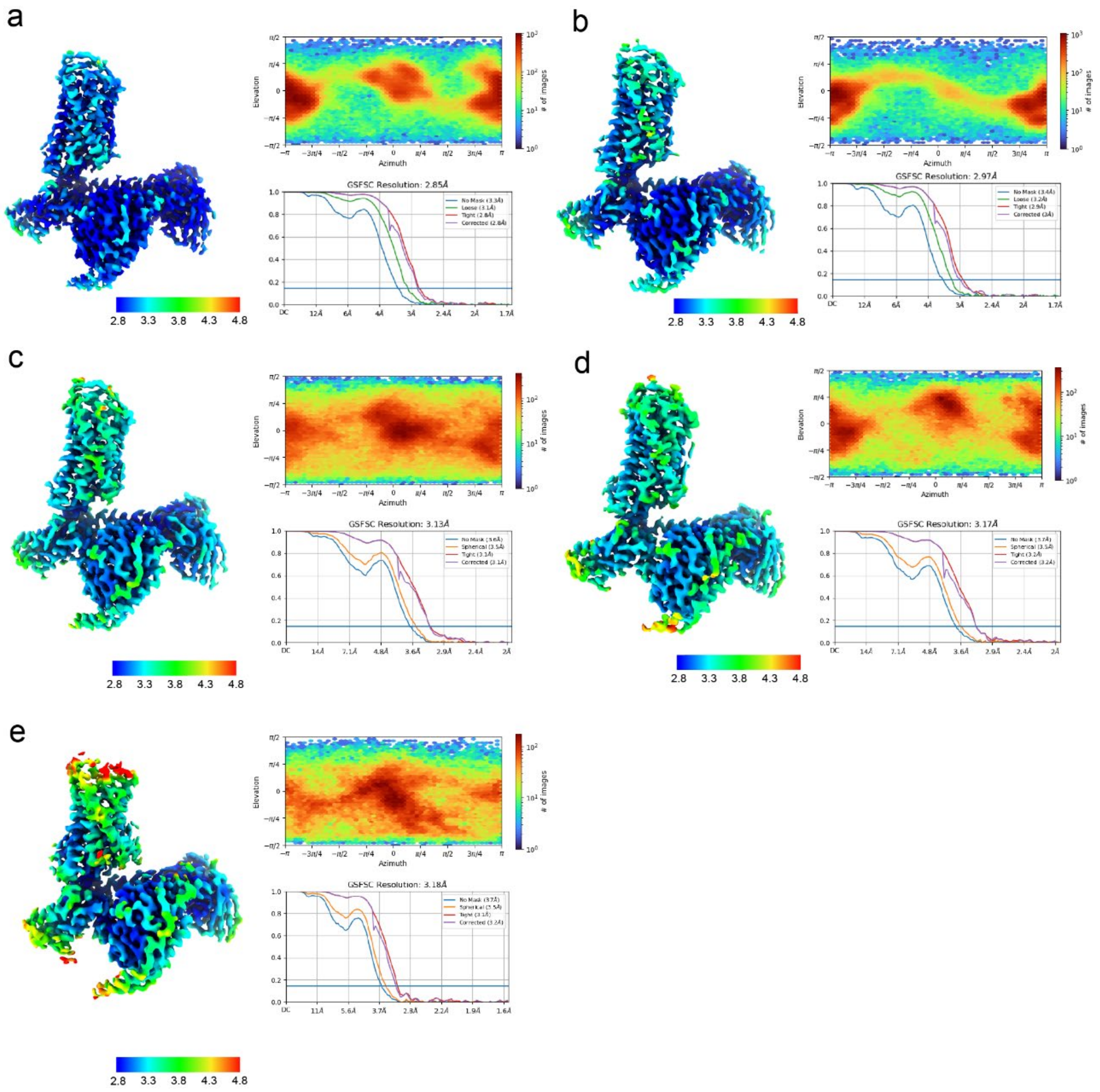

Supplementary Fig. 5 | Resolutions of the HCA2-Gi and HCA3-Gi complexes

a-e Local resolution analyses (left panels) of HCA2-Gi complexes with GSK256073 (a), MK6892 (b), LUF6283 (c), acifran (d), and the HCA3-Gi complex with acifran (e). Angular distribution plots and gold-standard Fourier shell correlation (GSFSC) curves (right panels) for the HCA2-Gi and HCA3-Gi complexes. Resolutions were estimated based on the FSC = 0.143 criterion.

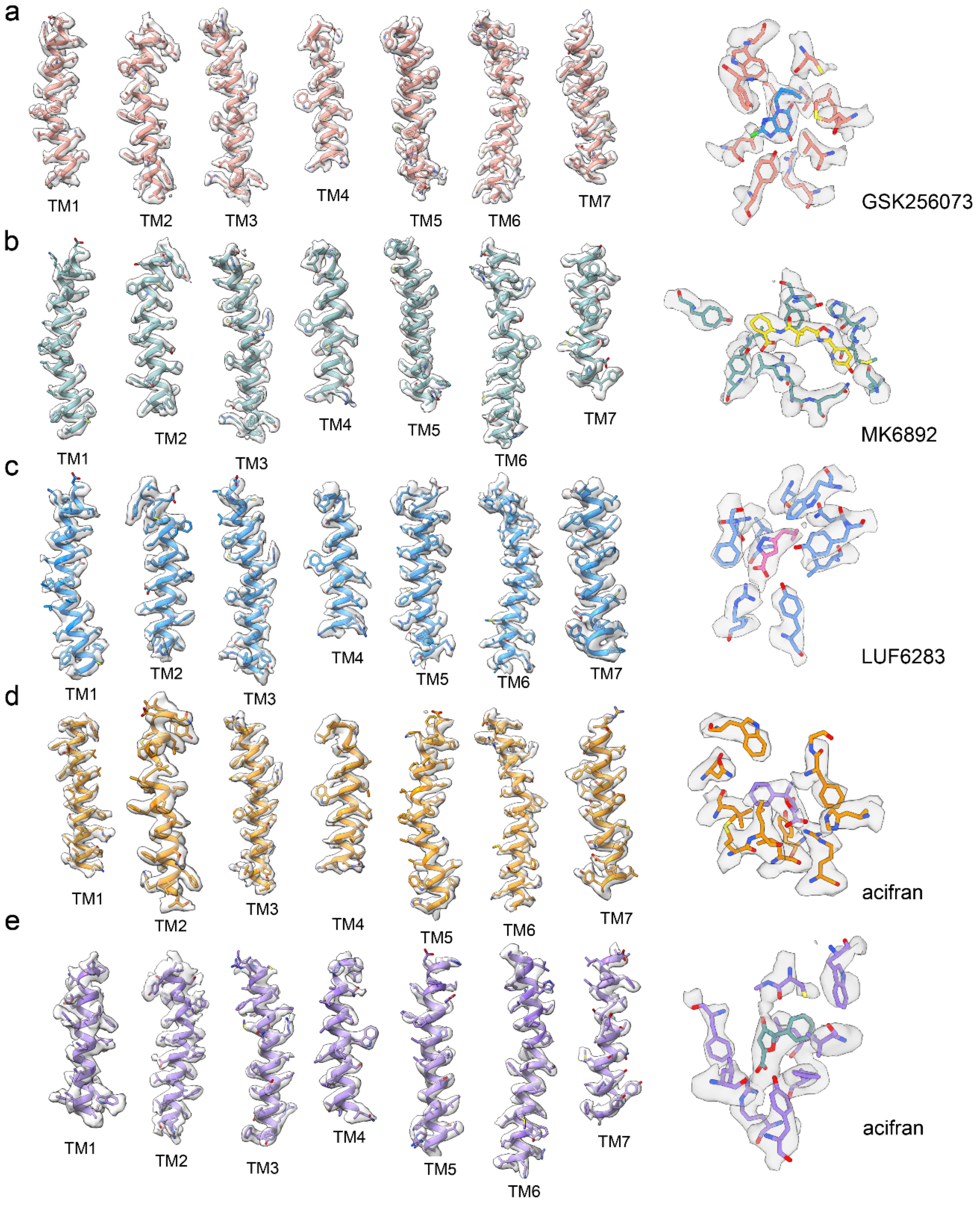

Supplementary Fig. 6 | Representative EM density maps

a-e Cryo-EM density maps at the 7 transmembrane helices and the residues involved in ligand binding of HCA2 with GSK256073 (a), MK6892 (b), LUF6283 (c), acifran (d), and HCA3 with acifran (e).

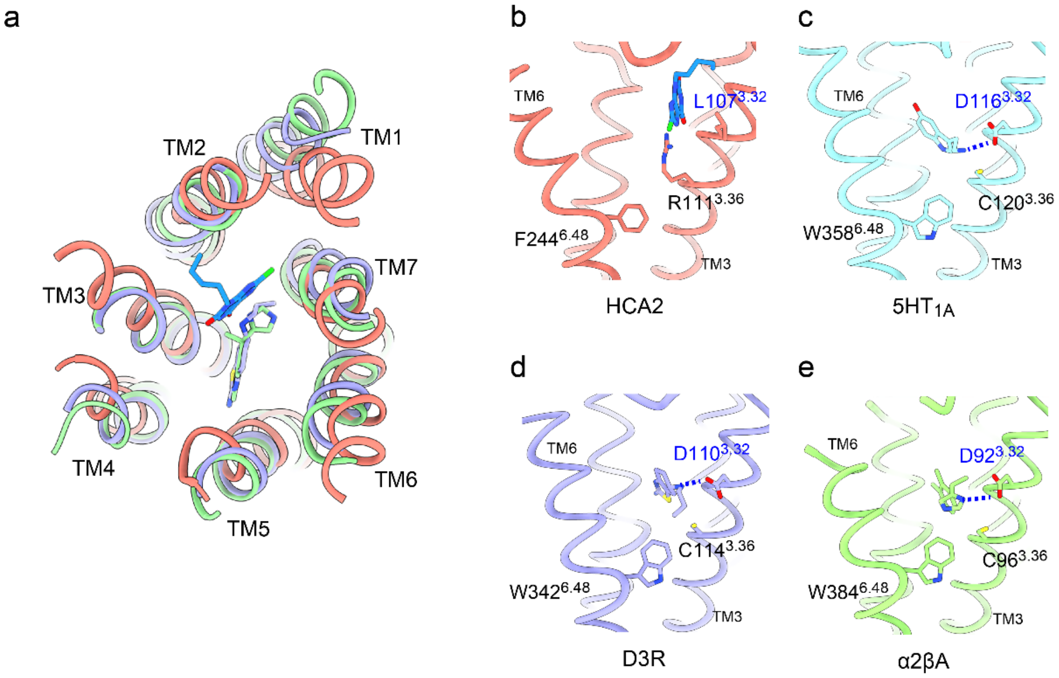

Supplementary Fig. 7 | Structural comparison of amine-coupled receptors

a Superimposed structures of HCA2 and amine-coupled receptors. Structural models of HCA2 (b, orange), 5-HT1AR (c, blue, PDB: 7E2Y), D3R (d, purple, PDB: 7CMU), and α2BAR (e, green, PDB: 6K41) are indicated by cartoon representation. All ligands are shown as stick models. **b-e** Ligand binding sites and residues at position 3.32 in HCA2 are compared with those in the active structures of HCA2, 5-HT1AR, D3R, α2BAR (e, PDB: 6K41). Colors of structural models are consistent with those in **a.**

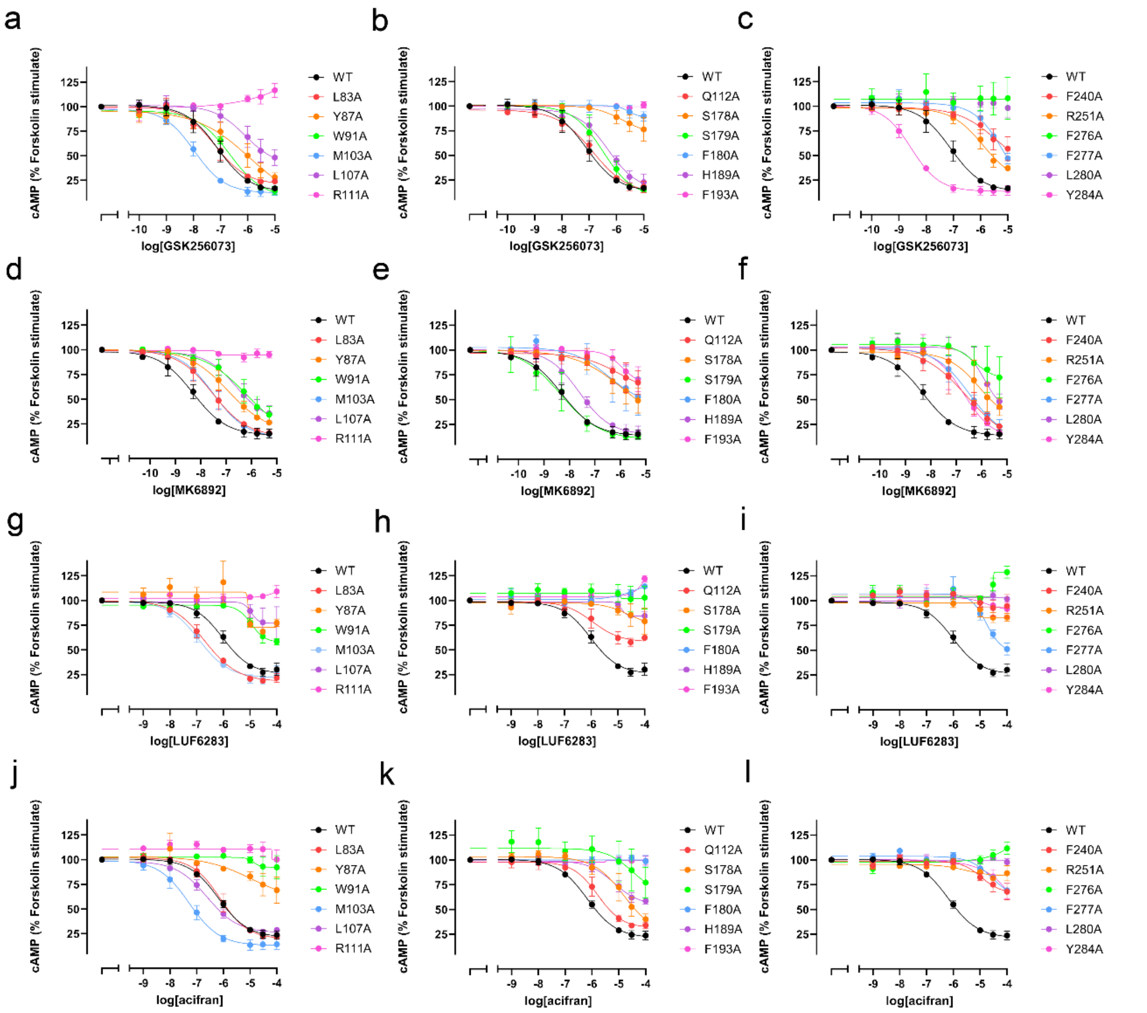

Supplementary Fig. 8 | Mutational analysis by ligand-induced cAMP inhibition assay

a-l Inhibition of cAMP production after stimulation was monitored in HEK293 cells transfected with WT and mutant constructs of HCA2 by applying GSK256073 **(a-c)**, MK6892 **(d-f)**, LUF6283 **(g-i)**, and acifran **(j-l)**. The count of each well was normalized to that of wells treated with forskolin alone, which was defined as 100%. Each point represents the mean ± SEM from 3 independent experiments (n=3). Source data are provided in the Source Data file.

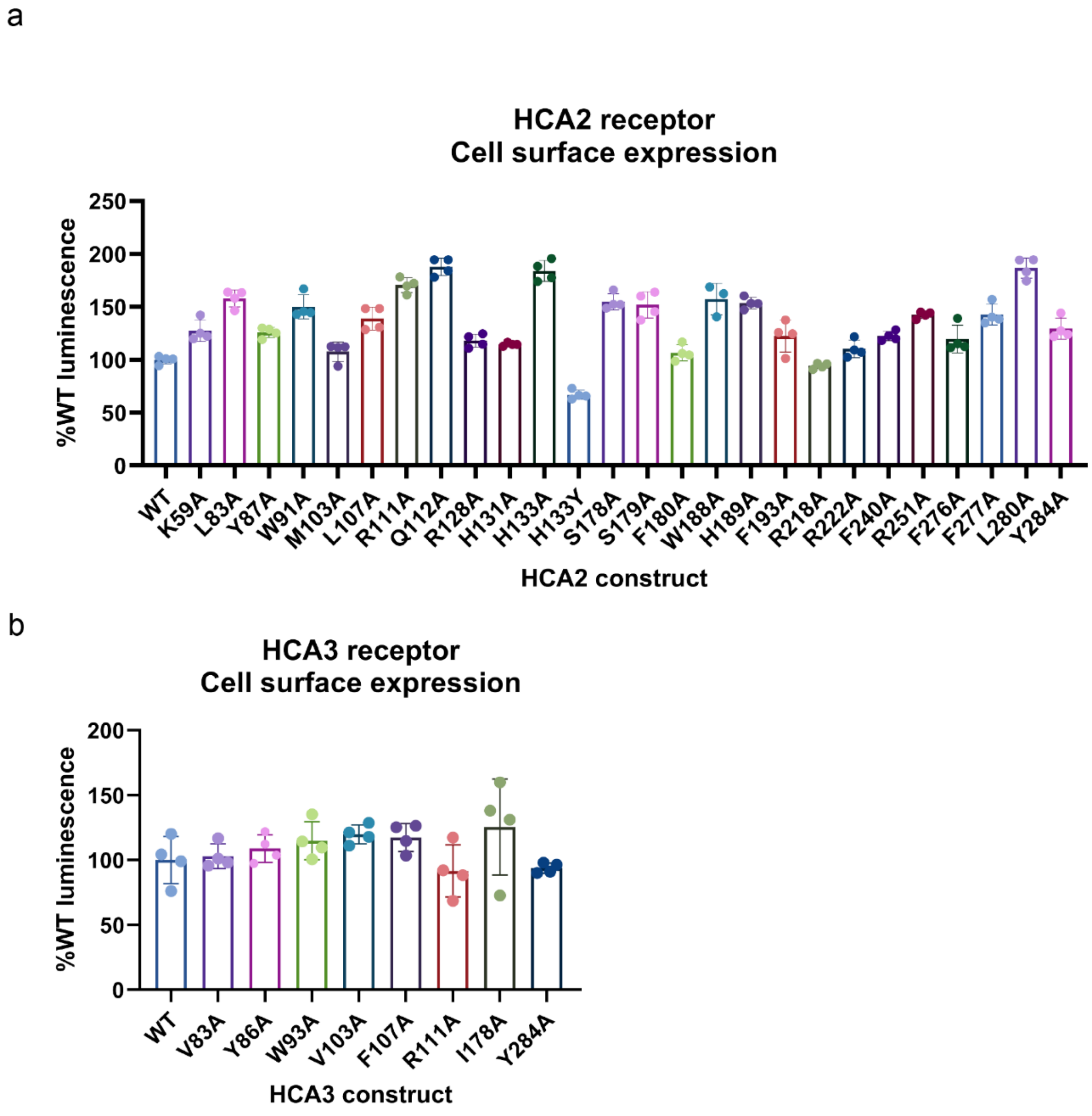

Supplementary Fig. 9 | Measurement of the cell surface expression level of wild-type and mutant HCA2 **(a)** and HCA3 **(b)** constructs

HEK-293 cells were used to express each construct and the expression levels were measured by ELISA. Data are mean ± SEM from 3~4 independent experiments (n=3~4).

	3x21	3x22	3x23	3x24	3x25	3x26	3x27	3x28	3x29	3x30	3x31	3x32	3x33	3x34	3x35	3x36	3x37	3x38	3x39	3x40	3x41	3x42	3x43	3x44	3x45	3x46	3x47	3x48	3x49	3x50	3x51	3x52	3x53	3x54	3x55	3x56
HCA1	G	D	I	P	C	R	V	G	L	F	T	L	A	M	N	R	A	G	S	I	V	F	L	T	V	V	A	A	D	R	Y	F	K	V	V	H
HCA2	G	D	I	P	C	R	L	M	L	F	M	L	A	M	N	R	Q	G	S	I	I	F	L	T	V	V	A	V	D	R	Y	F	R	V	V	H
HCA3	G	D	I	P	C	R	L	V	L	F	M	F	A	M	N	R	Q	G	S	I	I	F	L	T	V	V	A	V	D	R	Y	F	R	V	V	H
OXE1	G	A	A	A	C	K	V	N	L	F	M	L	S	T	N	R	T	A	S	V	V	F	L	T	A	I	A	L	N	R	Y	L	K	V	V	Q
GPR31	G	R	V	G	C	W	A	L	H	F	L	L	D	L	S	R	S	V	G	M	A	F	L	A	A	V	A	L	D	R	Y	L	R	V	V	H
GPR35	-	-	-	L	C	Q	L	S	Q	G	I	Y	L	T	N	R	Y	M	S	I	S	L	V	T	A	I	A	V	D	R	Y	V	A	V	R	H
SUCR	G	D	V	L	C	I	S	N	R	Y	V	L	H	A	N	L	Y	T	S	I	L	F	L	T	F	I	S	I	D	R	Y	L	I	I	K	Y
FFAR1	P	A	S	L	C	P	V	F	A	V	A	H	F	F	P	L	Y	A	G	G	G	F	L	A	A	L	S	A	G	R	Y	L	G	A	A	F
FFAR2	P	K	V	V	C	A	L	T	S	F	G	F	Y	S	S	I	Y	C	S	T	W	L	L	A	G	I	S	I	E	R	Y	L	G	V	A	F
FFAR3	P	F	I	L	C	P	L	S	G	F	I	F	F	T	T	I	Y	L	T	A	L	F	L	A	A	V	S	I	E	R	F	L	S	V	A	H
FFAR4	G	P	V	A	C	H	L	L	F	Y	V	M	T	L	S	G	S	V	T	I	L	T	L	A	A	V	S	L	E	R	M	V	C	I	V	H
BLT1	G	L	A	G	C	R	L	C	H	Y	V	C	G	V	S	M	Y	A	S	V	L	L	I	T	A	M	S	L	D	R	S	L	A	V	A	R
BLT2	G	Q	A	G	C	K	A	V	Y	Y	V	C	A	L	S	M	Y	A	S	V	L	L	T	G	L	L	S	L	Q	R	C	L	A	V	T	R
CysLT1	G	D	F	L	C	R	L	S	T	Y	A	L	Y	V	N	L	Y	C	S	I	F	F	M	T	A	M	S	F	F	R	C	I	A	I	V	F
CysLT2	G	D	L	A	C	R	I	M	S	Y	S	L	Y	V	N	M	Y	S	S	I	Y	F	L	T	V	L	S	V	V	R	F	L	A	M	V	H
CB1	S	R	N	V	F	L	F	K	L	G	G	V	T	A	S	F	T	A	S	V	G	S	L	F	L	T	A	I	D	R	Y	I	S	I	H	R
CB2	S	K	A	V	F	L	L	K	I	G	S	V	T	M	T	F	T	A	S	V	G	S	L	L	L	T	A	I	D	R	Y	L	C	L	R	Y
S1P1	T	P	A	Q	W	F	L	R	E	G	S	M	F	V	A	L	S	A	S	V	F	S	L	L	A	I	A	I	E	R	Y	I	T	M	L	K
LPA1	T	V	S	T	W	L	L	R	Q	G	L	I	D	T	S	L	T	A	S	V	A	N	L	L	A	I	A	I	E	R	H	I	T	V	F	R
LPA2	S	L	E	G	W	F	L	R	Q	G	L	L	D	T	S	L	T	A	S	V	A	T	L	L	A	I	A	V	E	R	H	R	S	V	M	A
LPA3	T	V	N	R	W	F	L	R	Q	G	L	L	D	S	S	L	T	A	S	L	T	N	L	L	V	I	A	V	E	R	H	M	S	I	M	R
LPA4	G	D	T	L	C	K	I	S	G	T	A	F	L	T	N	I	Y	G	S	M	L	F	L	T	C	I	S	V	D	R	F	L	A	I	V	Y
LPA5	P	D	L	L	C	Q	T	T	G	A	I	F	Q	M	N	M	Y	G	S	C	I	F	L	M	L	I	N	V	D	R	Y	A	A	I	V	H
LPA6	G	D	L	L	C	K	I	S	V	M	L	F	Y	T	N	M	Y	G	S	I	L	F	L	T	C	I	S	V	D	R	F	L	A	I	V	Y
5-HT1A	G	Q	V	T	C	D	L	F	I	A	L	D	V	L	C	C	T	S	S	I	L	H	L	C	A	I	A	L	D	R	Y	W	A	I	T	D
5-HT1B	G	Q	V	V	C	D	F	W	L	S	S	D	I	T	C	C	T	A	S	I	L	H	L	C	V	I	A	L	D	R	Y	W	A	I	T	D
5-HT2A	P	S	K	L	C	A	V	W	I	Y	L	D	V	L	F	S	T	A	S	I	M	H	L	C	A	I	S	L	D	R	Y	V	A	I	Q	N
5-HT2B	P	L	V	L	C	P	A	W	L	F	L	D	V	L	F	S	T	A	S	I	M	H	L	C	A	I	S	V	D	R	Y	I	A	I	K	K
D1	-	G	S	F	C	N	I	W	V	A	F	D	I	M	C	S	T	A	S	I	L	N	L	C	V	I	S	V	D	R	Y	W	A	I	S	S
D2	S	R	I	H	C	D	I	F	V	T	L	D	V	M	M	C	T	A	S	I	L	N	L	C	A	I	S	I	D	R	Y	T	A	V	A	M
GPBAR	G	Y	W	S	C	L	L	V	Y	L	A	P	N	F	S	F	L	S	L	L	A	N	L	L	L	V	H	G	E	R	Y	M	A	V	L	R
PAFR	P	K	F	L	C	N	V	A	G	C	L	F	F	I	N	T	Y	C	S	V	A	F	L	G	V	I	T	Y	N	R	F	Q	A	V	T	R

Supplementary Fig. 10 | Multiple sequence alignment of class A GPCRs

Sequence alignment of amino acid residues in TM3 of HCA receptors and other class A GPCRs. Residues at position 3.36 are indicated by the red box. Alignment data were obtained from the GPCRdb (gpcrdb.org).

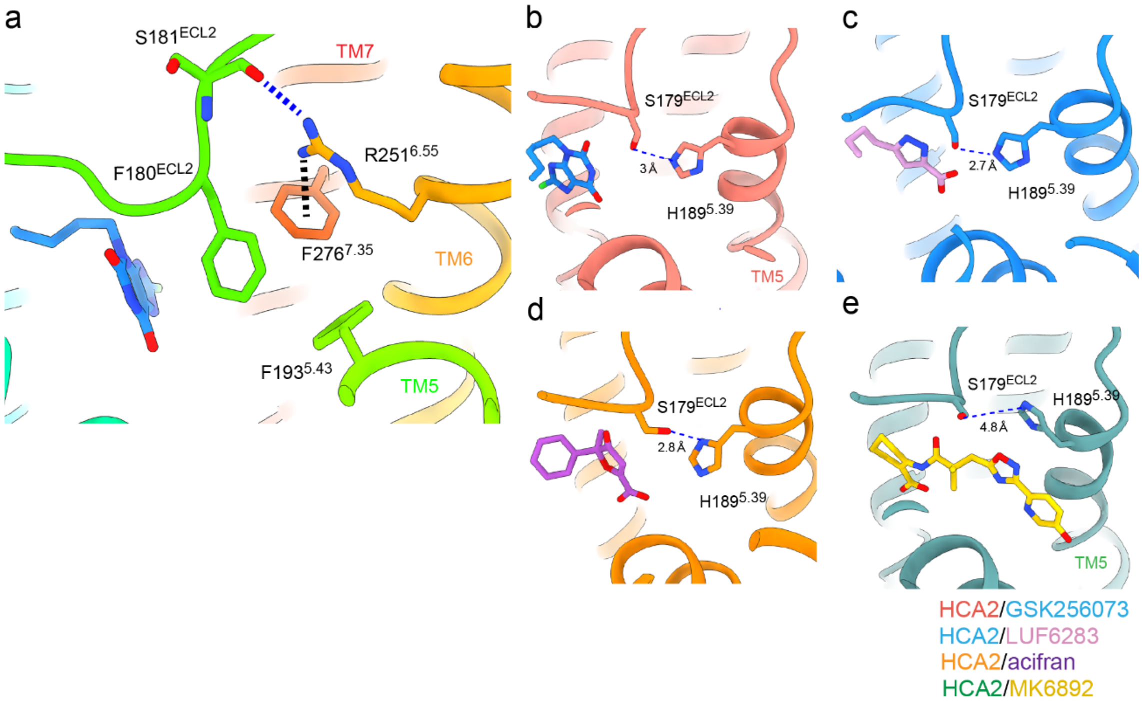

Supplementary Fig. 11 | Indirect interactions essential for ligand pocket formation

a R251$^{6.55}$ and a hydrophobic cluster composed of F180^{ECL2}, F193$^{5.43}$, and F276$^{7.35}$ in HCA2 are important for stabilizing the ligand binding pocket. The hydrogen bond is indicated by a blue dashed line and the π-cation interaction is indicated by a black dashed line. **b-e** Hydrogen bond between S179 and H189. All ligands and focused residues are shown by the stick models.

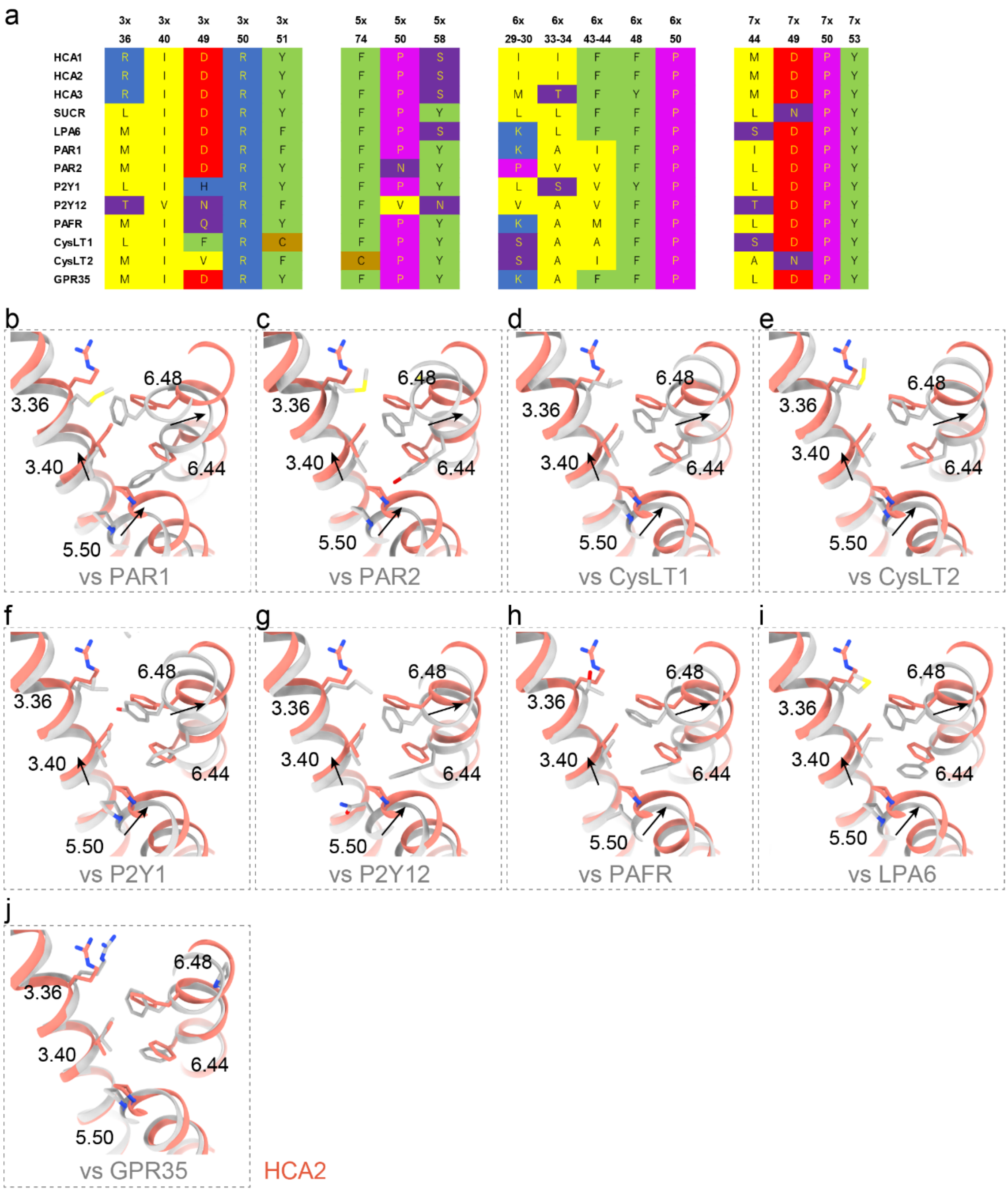

Supplementary Fig. 12 | Structural comparison of δ-branch GPCRs focused on the micro-switch

a Sequence alignment of amino acid residues focused on the conserved microswitch of δ-branch class A GPCRs. **b-j** Structural comparison of HCA2 (orange) with other δ-branch class A GPCRs (gray), including PAR1 (PDB: 3VW7)**(b)**, PAR2 (PDB: 5NDD)**(c)**, CysLT1 (PDB: 6RX5)**(d)**, CysLT2 (PDB: 6RZ6)**(e)**, P2Y1 (PDB: 4XNW)**(f)**, P2Y12 (PDB: 4PXZ)**(g)**, PAFR (PDB: 4PXZ)**(h)**, LPA6 (PDB: 5XSZ)**(i)**, and GPR35 (PDB : 8H8J)**(j)**

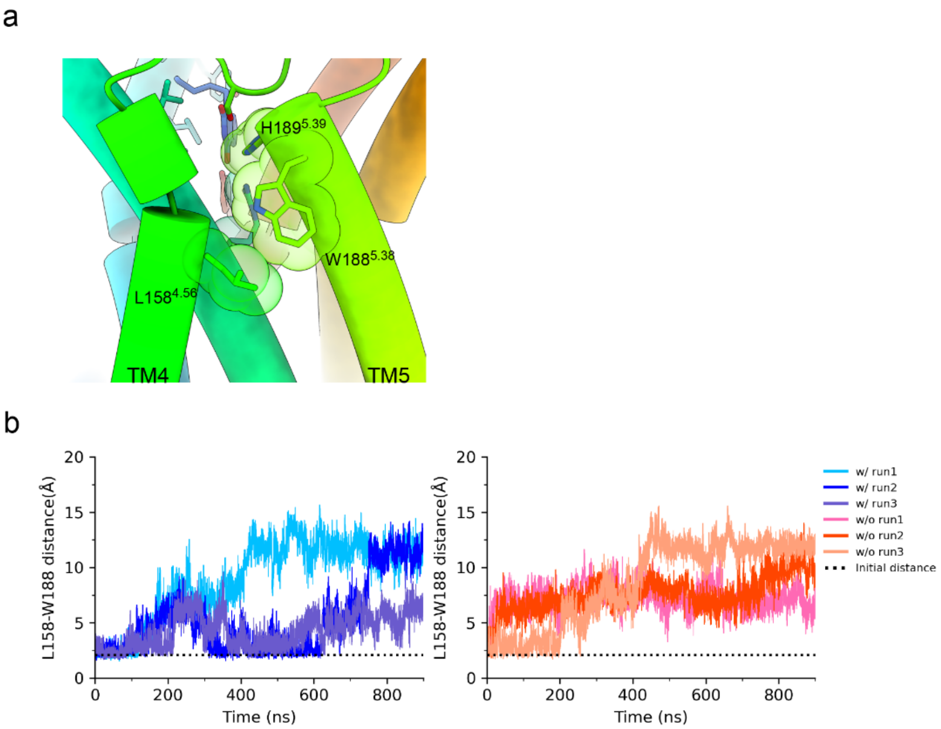

Supplementary Fig. 13 | Lateral gate and MD simulation

a Cross section and focused view of the lateral gate in HCA2 are shown. The amino acids of the side chain near the gate are indicated by a stick and ball model. **b** Molecular dynamics (MD) simulations in the presence (left) and absence (right) of GSK256073. Temporal changes in the minimum distance between all the atoms of L158$^{4.56}$ and W188$^{5.38}$ in the presence (left) and absence (right) of GSK256073 are shown. Simulations were performed in 3 independent runs in the presence and absence of GSK266073, respectively.

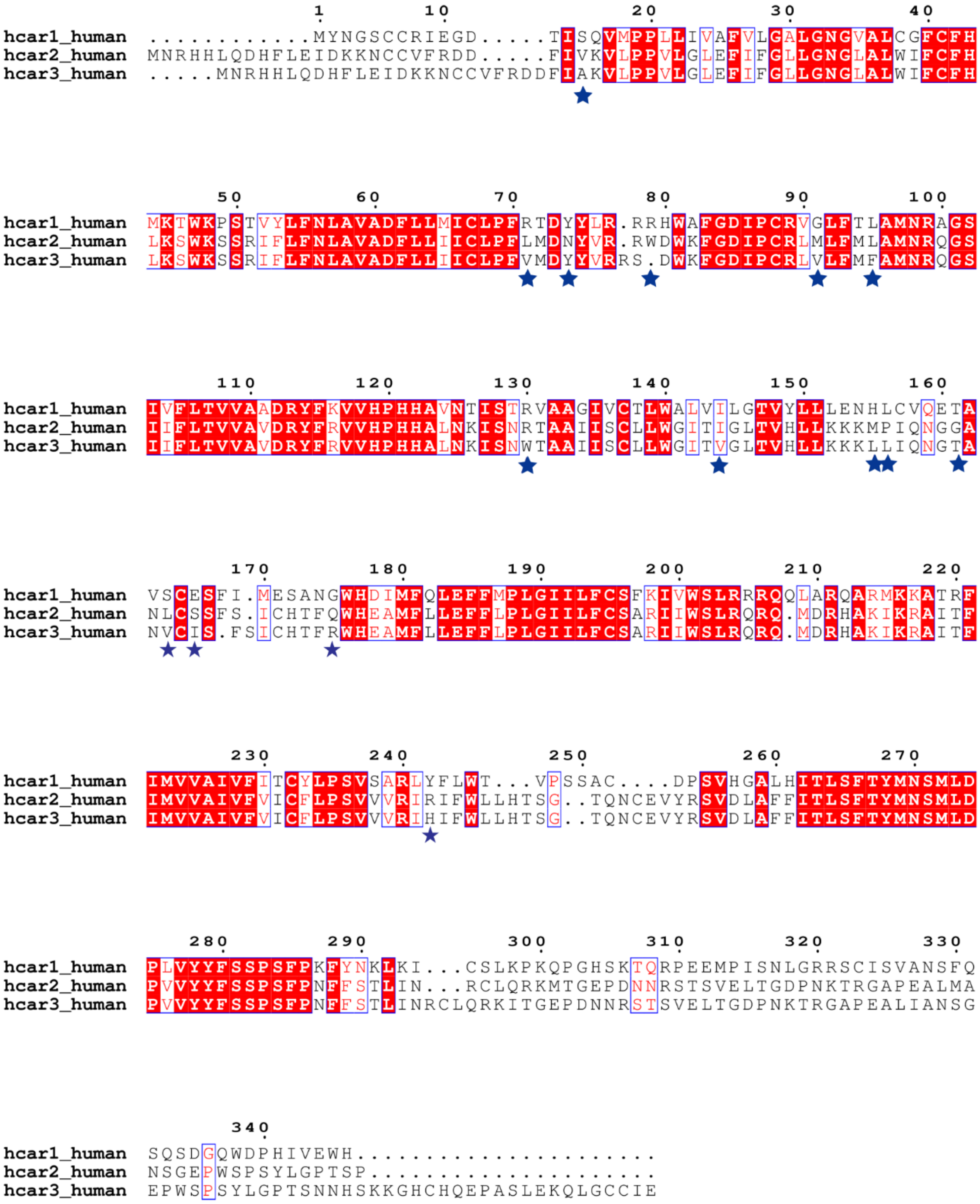

Supplementary Fig. 14 | Sequence alignment of the HCA receptors

Sequence alignment was prepared in GPCRdb (https://gpcrdb.org/), and the graphic was generated by the ESPript 3.0 server (https://espript.ibcp.fr/ESPript/). Differences in residues between HCA2 and HCA3 are indicated by blue stars.

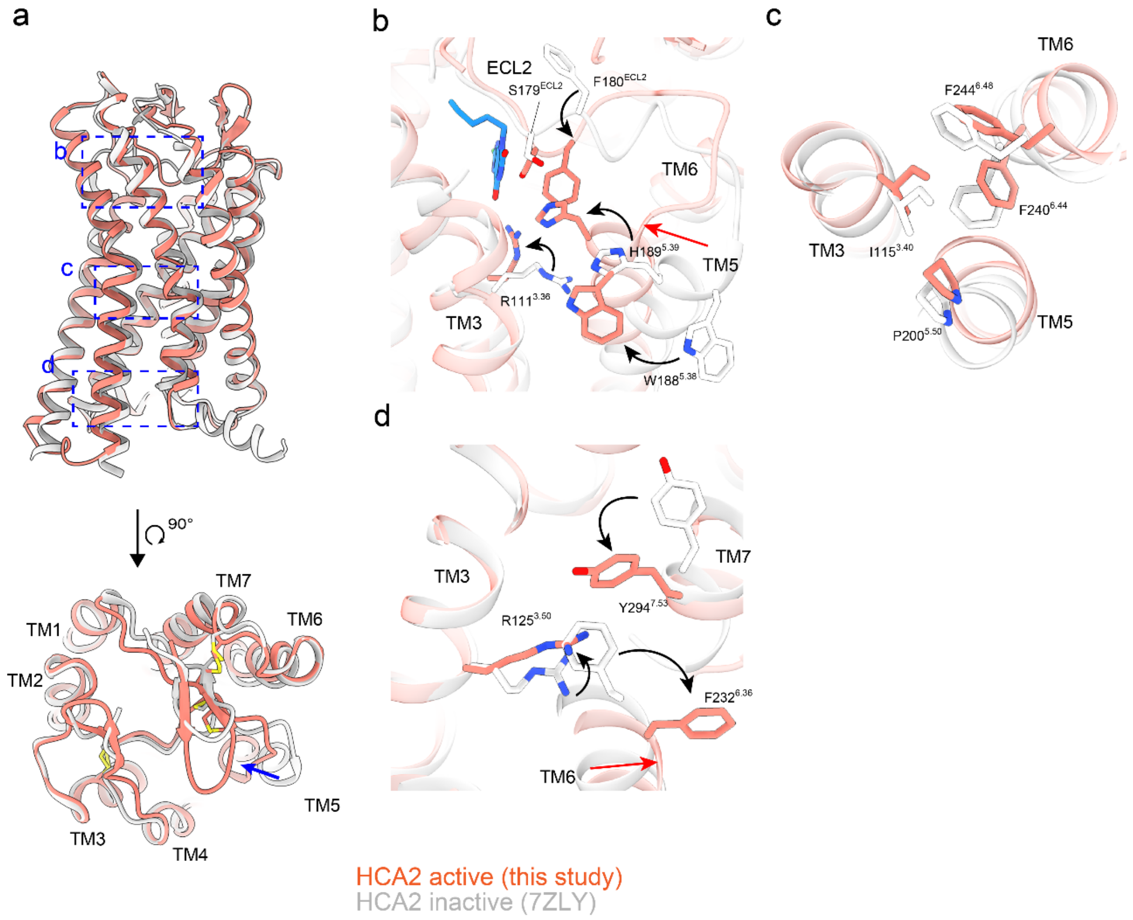

Supplementary Fig. 15 | Structural comparison with the inactive HCA2 structure

a Comparison of the active HCA2 structure with the inactive HCA2 structure (PDB: 7ZLY). Blue arrows indicate differences in the positions of TM5. **b** Close-up view of the extracellular side. Side chains revealing conformational differences between the 2 structures are shown in stick representation. **c** Conformations at the PIF motif. **d** Close-up view of the cytoplasmic side. Conformational changes of $R^{3.50}$ (DRY motif) and $Y^{7.53}$ (NPxxY motif) show a transition to the activated state.

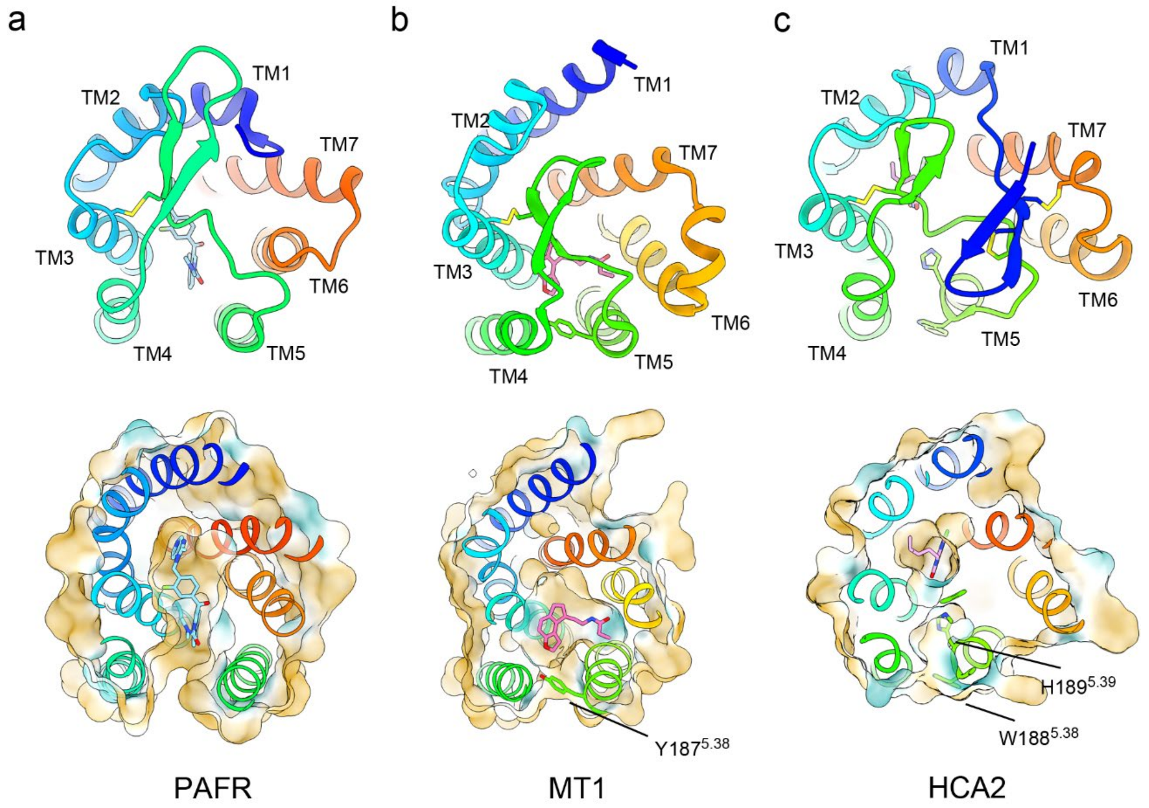

Supplementary Fig. 16 | Structural comparison of class A GPCR extracellular side and supposed ligand entrance

a Top view of PAFR (PDB: 5ZKQ) (a), MT1 (PDB: 7VGZ) (b), and HCA2 (c) in cartoon representation (upper) superimposed hydrophobic surface representation (lower). Cysteine residues forming disulfide bonds and ligands are represented by stick models. The side chain of amino acids in the ligand entrance is represented by a stick model.

Supplementary Table 1 Validation of Cryo-EM analysis

Cryo-EM data collection, refinement, and validation statistics

	HCA2-GSK256073 (EMDB-35442) (PDB 8IHB)	HCA2-MK6892 (EMDB-35443) (PDB 8IHF)	HCA2-LUF6283 (EMDB-35444) (PDB 8IHH)	HCA2-acifran (EMDB-35445) (PDB 8IHI)	HCA3-acifran (EMDB-35446) (PDB 8IHJ)	HCA3-acifran (local) (EMDB-35447) (PDB 8IHK)
**Data collection and processing**						
Magnification	105K	105K	50K	50K	60K	
Voltage (kV)	300	300	300	300	300	
Electron exposure (e–/Å^2)	49	49	69.6	69.6	71.2	
Defocus range (μm)	-0.7 to -1.5	-0.7 to -1.5	-1.0 to -2.0	-1.0 to -2.0	-1.0 to -2.0	
Pixel size (Å)	0.83	0.83	0.99	0.99	0.78	
Final particle images (no.)	245,517	181,958	181,273	146,577	95,567	
Map resolution (Å)	2.85	2.97	3.13	3.17	3.18	3.33
FSC threshold	0.143	0.143	0.143	0.143	0.143	0.143
Map resolution range (Å)	2.8-4.8	2.8-4.8	2.8-4.8	2.8-4.8	2.8-4.8	2.8-4.8
**Refinement**						
Model resolution (Å)	3.0	3.1	3.2	3.2	3.3	3.6
	0.5	0.5	0.5	0.5	0.5	0.5
FSC threshold						
Model resolution range (Å)	n/a	n/a	n/a	n/a	n/a	n/a
Map sharpening *B* factor (Å^2)	-96.8	-97.4	-87.8	-87.3	-94.5	-100.4
Model composition						
Non-hydrogen atoms	8709	8702	8649	8672	8510	2253
	1131	1131	1131	1131	1122	283
Protein residues	1	1	1	1	1	1

Ligands						
R.m.s. deviations						
Bond lengths (Å)	0.004	0.003	0.002	0.003	0.002	0.002
Bond angles (°)	0.6151	0.495	0.519	0.522	0.463	0.509
Validation						
MolProbity score	1.59	1.59	1.60	1.44	1.50	1.37
Clashscore	6.50	5.98	6.35	5.72	5.55	3.80
Poor rotamers	0.11	0.11	0.11	0.22	0.11	0.00
(%)						
Ramachandran						
plot	96.50	96.14	96.32	97.31	96.75	96.80
Favored (%)	3.50	3.86	3.68	2.69	3.25	3.2
Allowed (%)	0	0	0	0	0	0
Disallowed (%)						

Supplementary Table 2. primer list

Name	Sequence
hHCA2-L83A-Forward	CCATTCGCAATGGATAATTACGTTAGA
hHCA2-L83A-Reverse	ATCCATTGCGAATGGCAAACAGATGAT
hHCA2-Y87A-Forward	GATAATGCAGTTAGACGCTGGGACTGG
hHCA2-Y87A-Reverse	TCTAACTGCATTATCCATCAGGAATGGC
hHCA2-W91A-Forward	AGACGCGCAGACTGGAAGTTCGGTGA
hHCA2-W91A-Reverse	CCAGTCTGCGCGTCTAACGTAATTATCC
hHCA2-M103A-Forward	CGCTTGGCACTGTTCATGCTGGCCAT
hHCA2-M103A-Reverse	GAACAGTGCCAAGCGGCAGGGAATGTC
hHCA2-L107A-Forward	TTCATGGCAGCCATGAATAGGCAAGGA
hHCA2-L107A-Reverse	CATGGCTGCCATGAACAGCATCAAGCG
hHCA2-R111A-Forward	ATGAATGCACAAGGATCCATTATCTTCC
hHCA2-R111A-Reverse	TCCTTGTGCATTCATGGCCAGCATGA
hHCA2-Q112A-Forward	AATAGGGCAGGATCCATTATCTTCCTT
hHCA2-Q112A-Reverse	GGATCCTGCCCTATTCATGGCCAGCA
hHCA2-S178A-Forward	TTGTGTGCATCATTTTCAATCTGCCACA
hHCA2-S178A-Reverse	AAATGATGCACACAAGTTCGCGCCG
hHCA2-S179A-Forward	TGTAGCGCATTTTCAATCTGCCACAC
hHCA2-S179A-Reverse	TGAAAATGCGCTACACAAGTTCGCGC
hHCA2-F180A-Forward	AGCTCAGCATCAATCTGCCACACGTT
hHCA2-F180A-Reverse	GATTGATGCTGAGCTACACAAGTTCGCG
hHCA2-W188A-Forward	TTCCAAGCACATGAGGCCATGTTCCT
hHCA2-W188A-Reverse	CTCATGTGCTTGGAACGTGTGGCAGAT
hHCA2-H189A-Forward	CAATGGGCAGAGGCCATGTTCCTGCT
hHCA2-H189A-Reverse	GGCCTCTGCCCATTGGAACGTGTGGC
hHCA2-F193A-Forward	GCCATGGCACTGCTGGAGTTCTTCCTC
hHCA2-F193A-Reverse	CAGCAGTGCCATGGCCTCATGCCATTG
hHCA2-F240A-Forward	ATCGTGGCAGTCATATGTTTCCTGCC
hHCA2-F240A-Reverse	TATGACTGCCACGATAGCGACCACCA
hHCA2-R251A-Forward	GTTGTCGCAATCCGTATCTTCTGGCT
hHCA2-R251A-Reverse	ACGGATTGCGACAACCACGCTGGGCA
hHCA2-F232A-Forward	ATAACCGCAATCATGGTGGTCGCTATC
hHCA2-F232A-Reverse	CATGATTGCGGTTATTGCTCTCTTGATC

hHCA2-F276A-Forward	CTGGCGGCATTCATTACTCTCTCATT
hHCA2-F276A-Reverse	AATGAATGCCGCCAGGTCAACGCTCC
hHCA2-F277A-Forward	CGTTCGCAATTACTCTCTCATTTACGT
hHCA2-F277A-Reverse	AGTAATTGCGAACGCCAGGTCAACGC
hHCA2-L280A-Forward	ATTACTGCATCATTTACGTATATGAACT
hHCA2-L280A-Reverse	AAATGATGCAGTAATGAAGAACGCC
hHCA2-Y284A-Forward	TTTACGGCAATGAACTCCATGTTGGA
hHCA2-Y284A-Reverse	GTTCATTGCCGTAAATGAGAGAGTAATG
hHCA3-V83A-Forward	CCATTCGCAATGGACTACTACGTGCG
hHCA3-V83A-Reverse	GTCCATTGCGAATGGCAAACAGATGA
hHCA3-Y86A-Forward	ATGGACGCATACGTGCGTCGTTCAGA
hHCA3-Y86A-Reverse	CACGTATGCGTCCATGACGAATGGCA
hHCA3-W93A-Forward	TCAGACGCAAAGTTCGGTGACATTCC
hHCA3-W93A-Reverse	GAACTTTGCGTCTGAACGACGCACGT
hHCA3-V103A-Forward	CGCTTGGCACTGTTCATGTTCGCCAT
hHCA3-V103A-Reverse	GAACAGTGCCAAGCGGCAGGGAATGT
hHCA3-L104A-Forward	TTGGTGGCATTCATGTTCGCCATGAAT
hHCA3-L104A-Reverse	CATGAATGCCACCAAGCGGCAGGGAA
hHCA3-F107A-Forward	TTCATGGCAGCCATGAATAGGCAAGGA
hHCA3-F107A-Reverse	CATGGCTGCCATGAACAGCACCAAGC
hHCA3-R111A-Forward	ATGAATGCACAAGGATCCATTATCTTCCT
hHCA3-R111A-Forward	TCCTTGTGCATTCATGGCGAACATGA
hHCA3-I178A-Forward	GTGTGTGCATCATTTTCAATCTGCCACA
hHCA3-I178A-Forward	AAATGATGCACACACGTTGGCCGTACC
hHCA3-Y284A-Forward	TTTACGGCAATGAACTCCATGTTGGACC
hHCA3-Y284A-Forward	GTTCATTGCCGTAAATGAGAGAGTAATG A
