# Supplementary material for: Structural basis of hydroxycarboxylic acid receptor signaling mechanisms through ligand binding
Source: Nat Commun. 2023 Sep 22;14:5899. doi: 10.1038/s41467-023-41650-7 (PMC10516952; doi:10.1038/s41467-023-41650-7)
Supplement: Supplementary file 3 — Reporting Summary [file 41467_2023_41650_MOESM3_ESM.pdf]

nature portfolio

Corresponding author(s): Yoshinori Fujiyoshi

Last updated by author(s): Aug 22, 2023

Reporting Summary

Nature Portfolio wishes to improve the reproducibility of the work that we publish. This form provides structure for consistency and transparency in reporting. For further information on Nature Portfolio policies, see our Editorial Policies and the Editorial Policy Checklist.

Statistics

For all statistical analyses, confirm that the following items are present in the figure legend, table legend, main text, or Methods section.

n/a	Confirmed	
☐	☒	The exact sample size (n) for each experimental group/condition, given as a discrete number and unit of measurement
☐	☒	A statement on whether measurements were taken from distinct samples or whether the same sample was measured repeatedly
☒	☐	The statistical test(s) used AND whether they are one- or two-sided<br>*Only common tests should be described solely by name; describe more complex techniques in the Methods section.*
☒	☐	A description of all covariates tested
☒	☐	A description of any assumptions or corrections, such as tests of normality and adjustment for multiple comparisons
☐	☒	A full description of the statistical parameters including central tendency (e.g. means) or other basic estimates (e.g. regression coefficient) AND variation (e.g. standard deviation) or associated estimates of uncertainty (e.g. confidence intervals)
☒	☐	For null hypothesis testing, the test statistic (e.g. F, t, r) with confidence intervals, effect sizes, degrees of freedom and P value noted<br>*Give P values as exact values whenever suitable.*
☒	☐	For Bayesian analysis, information on the choice of priors and Markov chain Monte Carlo settings
☒	☐	For hierarchical and complex designs, identification of the appropriate level for tests and full reporting of outcomes
☒	☐	Estimates of effect sizes (e.g. Cohen's d, Pearson's r), indicating how they were calculated

Our web collection on statistics for biologists contains articles on many of the points above.

Software and code

Policy information about availability of computer code

Data collection	Automated data collection on the Titan Krios and JEM-Z320FHC was performed using Serial EM version 3.8.9.
Data analysis	RELION4.0, PHENIX, COOT, UCSF chimeraX, pymol, Graphpad Prism 10, cryoSPARC, MotionCorr2.1, CTFFIND4.1, GROMACS, MDAnalysis 2.4.2

For manuscripts utilizing custom algorithms or software that are central to the research but not yet described in published literature, software must be made available to editors and reviewers. We strongly encourage code deposition in a community repository (e.g. GitHub). See the Nature Portfolio guidelines for submitting code & software for further information.

Data

Policy information about availability of data

All manuscripts must include a data availability statement. This statement should provide the following information, where applicable:

- Accession codes, unique identifiers, or web links for publicly available datasets
- A description of any restrictions on data availability
- For clinical datasets or third party data, please ensure that the statement adheres to our policy

Atomic coordinates for the HCA2-Gi complexes with GSK256073, MK6892, LUF6283, acifran and HCA3-Gi complexes with acifran have been deposited in the Protein Data Bank under accession code 8IHB, 8IHF, 8IHH, 8IHI, 8IHJ, and 8IHK, respectively. The associated electron microscopy data have been deposited in the Electron Microscopy Data Bank under accession code EMD-355442, EMD-35443, EMD-35444, EMD-35445, EMD-35446, EMD-35447. Sequence alignment was performed using the GPCRdb(https://gpcrdb.org/) and the representation of the sequence alignment was generated using the ESPript website (http://espript.ibcp.fr).

Research involving human participants, their data, or biological material

Policy information about studies with human participants or human data. See also policy information about sex, gender (identity/presentation), and sexual orientation and race, ethnicity and racism.

Reporting on sex and gender	N/A
Reporting on race, ethnicity, or other socially relevant groupings	N/A
Population characteristics	N/A
Recruitment	N/A
Ethics oversight	N/A

Note that full information on the approval of the study protocol must also be provided in the manuscript.

Field-specific reporting

Please select the one below that is the best fit for your research. If you are not sure, read the appropriate sections before making your selection.

☒ Life sciences ☐ Behavioural & social sciences ☐ Ecological, evolutionary & environmental sciences

For a reference copy of the document with all sections, see nature.com/documents/nr-reporting-summary-flat.pdf

Life sciences study design

All studies must disclose on these points even when the disclosure is negative.

Sample size	Sample sizes were determined based on prior literature and best practices in the field; no statistical methods were used to predetermine sample size. cAMP assay and BRET2 assay, and cell surface expression , at least three independent biological experiment (n=3) were performed as depicted in related Figure legends. Each biological replicate has two or three technical replicates. Data were analyzed by fitting various ligand concentration and readout using appropriate equations in Graphpad Prism 10.0.
Data exclusions	no data was excluded
Replication	All attempts at replication were successful.
Randomization	Animal experiments were not performed in this study, so no randomization was needed.
Blinding	Animal experiments were not performed in this study, so Investigators were not blinded to the experiment

Reporting for specific materials, systems and methods

We require information from authors about some types of materials, experimental systems and methods used in many studies. Here, indicate whether each material, system or method listed is relevant to your study. If you are not sure if a list item applies to your research, read the appropriate section before selecting a response.

Materials & experimental systems

n/a	Involved in the study	
☐	☒	Antibodies
☐	☒	Eukaryotic cell lines
☒	☐	Palaeontology and archaeology
☒	☐	Animals and other organisms
☒	☐	Clinical data
☒	☐	Dual use research of concern
☒	☐	Plants

Methods

n/a	Involved in the study	
☒	☐	ChIP-seq
☒	☐	Flow cytometry
☒	☐	MRI-based neuroimaging

Antibodies

Antibodies used	For structure determination, the single chain antibody scFv16 was used, as originally described by Koehl et al., Nature 558: 547

Antibodies used	(2018). The scFv16 used in this study was produced in Sf9 cells using a synthetic gene, as described in Methods. For measurement of receptor cell surface expression, monoclonal anti-FLAG M2-Peroxidase (HRP) antibody (sigma-Aldrich, Catalog #A8592)
Validation	The scFv16 sequence was validated by DNA sequencing of the expression vector and resulting baculovirus construct, as well as SOS PAGE analysis of the purified protein.<br>Monoclonal anti-FLAG M2-HRP antibody: https://www.sigmaaldrich.com/JP/ja/product/sigma/a8592

Eukaryotic cell lines

Policy information about cell lines and Sex and Gender in Research

Cell line source(s)	Sf+ cells were purchased from Protein Sciences Corporation (Now, Merck owns Licenses Proprietary).<br>Expi293 cells were purchased from Thermo Fisher Scientific.
Authentication	Early passages were used.
Mycoplasma contamination	Cell lines have not been authenticated Cell lines were not tested for mycoplasma contamination
Commonly misidentified lines<br>(See ICLAC register)	None of commonly misidentified lines were used in this study.
